# Immune Thrombocytopenic Purpura and Paradoxical Thrombosis: A Systematic Review of Case Reports

**DOI:** 10.7759/cureus.30279

**Published:** 2022-10-13

**Authors:** Elrazi A Ali, Maimoonah Rasheed, Anas Al-sadi, Abdalaziz M Awadelkarim, Eltaib A Saad, Mohamed A Yassin

**Affiliations:** 1 Internal Medicine, Interfaith Medical Center/One Brooklyn Health, Brooklyn, USA; 2 Internal Medicine, Hamad Medical Corporation, Doha, QAT; 3 Internal Medicine, Wayne State University Detroit Medical Center, Detroit, USA; 4 Internal Medicine, Saint Francis Hospital, Evanatson, USA; 5 Hematology and Oncology, Hamad General Hospital, Doha, QAT

**Keywords:** thrombocytopnia, werlhof’s disease, infarction, thrombosis, immune thrombocytopenic purpura

## Abstract

Background and Aims: Immune thrombocytopenic purpura (ITP) is an acquired bleeding disorder characterized by autoantibodies against platelets. The clinical presentation is variable; the main symptom is bleeding, and many patients are asymptomatic; others have nonspecific symptoms like fatigue. Uncommonly, ITP can present with paradoxical thrombosis. The risk of thrombosis in ITP may be higher than expected, which makes the management of ITP more challenging. This review aims to evaluate patients with ITP who develop thrombosis and identify potential risk factors related to thrombosis in this category of patients.

Materials and Methods: English literature was searched using the Preferred Reporting Items for Systematic Reviews and Meta-Analyses (PRISMA) guidelines for adults above 18 years with primary ITP who had infarctions or thrombotic events. Patients with secondary ITP were excluded. The search included articles published up to 20th October 2021.

Results: A total of 73 articles were included. Seventy-seven patients with ITP had developed infarctions and various thrombotic events. Sixty-three patients had arterial events, and 14 patients developed venous thrombotic events.

Conclusion: Patients with ITP have low platelets, which predispose them to bleed; despite that, serious thrombotic complications can happen in these patients and are difficult to predict. Therefore, it is critical for physicians to understand that ITP is paradoxically a prothrombotic condition and to address preventive thromboembolic measures whenever possible.

## Introduction and background

Immune thrombocytopenic purpura (ITP), previously known as Werlhof’s Disease, is a hematological disorder [1] characterized by immune-mediated destruction of platelets and persistently decreased platelet count (PLT); hence, the bleeding tendency is the hallmark of the disease. ITP can be either primary or secondary to another disease such as autoimmune disease, malignancies like chronic lymphocytic leukemia, or infections like human immunodeficiency virus (HIV) and hepatitis C virus (HCV) or post-vaccine [2]. The underlying mechanism for thrombocytopenia in ITP is not fully understood. The possible mechanism is autoantibodies targeting platelet surface glycoproteins, such as GPIIb/IIIa and GPIb/IX complexes [3]. The diagnosis of ITP is usually made after secondary causes have been ruled out by a thorough history, physical examination, and investigations. ITP usually presents with bleeding, which is seen in up to two-thirds of patients, but a significant number of patients are asymptomatic. Patients with significant bleeding usually have PLTs below 20,000/mL; however, the relation between the plate count and bleeding risk is unclear [3]. Recently, thromboembolic events have been increasingly reported in patients with ITP despite the low PLT [4]. The presence of thrombosis and infarction in patients with ITP is an unexpected finding that can change the concept of ITP and fill the gaps in our understanding of the disease. In this review, we tried to study the reported cases of the thromboembolic phenomenon in patients with ITP with respect to patient characteristics, disease, and hematologic parameters at the time of thrombosis to understand the risk factors and underlying mechanisms.

## Review

Methodology

Literature Search Strategy

**Figure 1:**
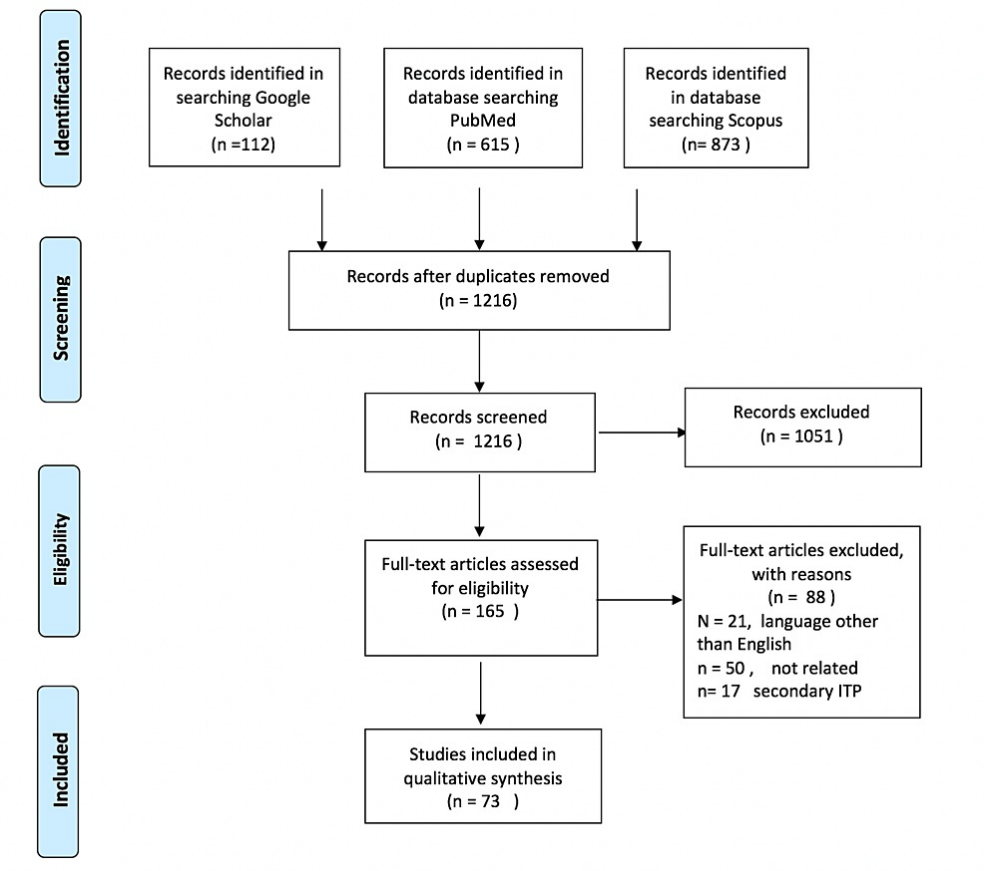
The PRISMA flow diagram detailing patients with immune thrombocytopenia purpura who developed thrombotic events or infarction. PRISMA, preferred reporting items for systematic reviews and meta-analyses

Following the Preferred Reporting Items for Systematic Reviews and Meta-Analyses (PRISMA) standards, we conducted a systematic review. A search of the English literature (PubMed, SCOPUS, and Google scholar) was conducted looking for articles describing thrombotic events in patients with ITP. We used the search terms: “immune thrombocytopenic purpura” with “thrombosis” and “infarction”. The reference in the included papers was scanned for any additional articles. The primary search process and secondary search process included articles published up to 20th October 2021. Thromboembolic events included were arterial thromboembolic events such as myocardial infarction, unstable angina, and ischemic stroke, and venous thromboembolic events like (pulmonary embolism, deep vein thrombosis, cerebral venous thrombosis, and portal vein thrombosis). A total of 73 articles were included (Figure 1).

Definitions

 According to the ITP International Working Group proposed definitions of disease [5]:

- Newly diagnosed ITP is a disease that was diagnosed within the past three months.

- Persistent ITP is a disease diagnosed for 3-12 months duration.

- Chronic ITP is a disease that lasts longer than a year.

Inclusion Criteria

- adult population age 18 years and above with ITP who developed thrombosis or infarction.

Exclusion Criteria

- Gray literature

- Reviews and cases with insufficient data

- Age less than 18 years

- Hemorrhagic infarctions

- Secondary thrombocytopenia: autoimmune diseases like systemic lupus erythematosus (SLE), HIV, HCV, cirrhosis, lymphoma…

- Patient with a prothrombotic condition like pregnancy, factor V laden, prothrombin mutation, and antiphospholipid syndrome

- Vaccine-related ITP

- Post-infectious or post coronavirus disease 2019 (COVID-19) related ITP

- Evan syndrome

Study Selection

Two independent reviewers examined the titles and abstracts of the records, excluding papers that did not meet our inclusion criteria. Inter-rater conflicts were settled with the help of a third reviewer and a discussion among the reviewers.

Data Extraction

Two reviewers extracted the date of publication, patient characteristics, treatment received, and the outcome.

Results

A total of 73 articles (Figure 1) reported 77 patients with ITP who developed thrombotic events or infarctions identified in Tables 1-2. Some 63 patients were with arterial events and 14 patients were with venous thrombotic events [6-78]. Some 44 patients had chronic ITP, one with persistent ITP, and 18 with a new diagnosis (less than three months), one patient with a recent diagnosis with no duration was specified, and others had ITP with no clear mention of onset or time of diagnosis. Some 38 patients were females, and 40 were males. The youngest patient was 18 years old, and the most senior was 83 years old. The mean age at the time of the event was 55.4 years, The mean age of venous thrombosis patients was 44.5 years, and the mean age of arterial thrombosis patients was 57.8 years. Thrombotic events affected different organs and locations; 14 patients had a stroke (infarction), 48 patients had coronary artery disease (ACS MI 3VD), one patient with cutaneous infarction, and one with mural aortic thrombus (Table 1). Some 14 patients had venous thrombotic events, including venous thrombosis in the central nervous system (venous sinus thrombosis) as well as thrombosis in the portal vein axillary brachial and jugular veins and intracardiac thrombus (Table 2). Treatment modalities used for ITP include patients, not on medication n=8, steroids n=39, danazol n=3, splenectomy n=10, one patient with splenic artery embolization, intravenous immune globulin (IVIG) n=13, platelet transfusion n=3, a thrombopoietin receptor agonist eltrombopag n=9, romiplostim n=3, rituximab n=1, and 17 patients with no mention of previous treatment.

**Table 1:** Characteristics of patients with ITP during the time of thrombosis. N/A, non-applicable; PCI, percutaneous coronary intervention; MI, myocardial infarction; 3VD, three-vessel disease; HTN, hypertension; DM, diabetes mellitus; DAP, dual antiplatelets; ASA, aspirin; CABG, coronary artery bypass grafting; UFH, unfractionated heparin; UC, ulcerative colitis; PE, pulmonary embolism; ITP, immune thrombocytopenic purpura; PLT, platelet count; PCI, percutaneous coronary intervention; PMH, past medical history

Reference	Age gender	PLT count at the time of event x109/L	Duration of ITP	Site of thrombosis	Treatment before the event	Intervention for thrombois	PMH or other comorbid condition	Outcome
[6]	46 F	20	New	Stroke	Non	Aspirin discontinued, started on dexamethasone	XXX syndrome (trisomy X (47,XXX karyotype))	Improved, discharged
[7]	80 M	15	2 months	MI	Danazol	PCI aspirin clopidogrel beta blockers fondaparinux	Mild aortic stenosis, HTN, DM 2	No events or bleeding complications
[8]	72 F	59	10 years	MI	Steroids	PCI	Angina and arteriosclerosis obliterans	Hematoma around the puncture side, discharged after three weeks
[9]	72 M	40	Chronic	MI	Steroids	CABG; seven units of platelets, transfused intraoperatively	N/A	Discharged
[9]	72 F	49	15 years	MI	Steroids intolerant	CABG, two consecutive days of IVIG	N/A	Discharged home stable
[9]	69 M	65	N/A	MI	Steroids intolerant	CABG, two days of IVIG therapy	N/A	Discharged
[10]	47 F	21	3 years	MI	Steroids	PCI, UFH, aspirin clopidogrel	DM	Discharged
[11]	37 M	39	20 years	MI	Not on medications	PCI, UFH, aspirin clopidogrel	None	Discharged. No bleeding complications in other locations.
[12]	77 M	70	30 years	MI	N/A	PCI, UFH, aspirin	HTN, hypercholesterolemia, CABG	Readmitted after five weeks with stenosis of the previous lesion
[13]	70 M	170	10 years	MI	Not on medications	PCI, aspirin clopidgrel abciximab	N/A	N/A
[14]	71 F	16	Chronic	MI	N/A	PCI	HTN, chronic refractory ITP	Stent patency at two years’ follow-up
[15]	37 F	568	N/A	Stroke	N/A	4 units of packed RBC, 24 units of platelets, and 4 units of FFP	RTA traumatic spleen rupture	Mild visual recovery
[16]	79 F	22	N/A	Stroke	Not on treatment	Prednisolone. IVIG. Aspirin then changed to clopidogrel, tapering the course of prednisolone	HTN	His speech has continued to improve
[17]	80 F	167	3 years	Stroke	Prednisolone and romiplostim	IVIG given and PLT reached 82	N/A	Hemorrhagic transformation, on day 33, she died of respiratory failure
[18]	75 M	18	8 years	MI	Non	PCI : cutting balloon then DAPT	CAD with stent to LAD five years back	One month later he developed new ACS, Restenosis, PCI was done again with DES to LAD
[19]	51 M	14	15	MI	Non	PCI with bare metal stent then DAPT	DM/HTN/dyslipidemia	Two weeks later, the patient was doing fine, with symptoms free
[20]	46 M	24	6	MI	Non	PCI : balloon angioplasty	DM/dyslipidemia/smoker	Discharged then underwent splenectomy
[21]	49M	1	New	MI	Non	Only supportive measure because it is secondary ischemia	Non	Discharged
[22]	49F	3	3 years	Stroke	Steroid / Azathioprine / Danazol	Supportive treatment	DM/HTN/hyperthyroidism	Death
[23]	50 M	658	8 years	MI	Steroid, Eltrombopag, Immunosuppressant, IVIG	Balloon angioplasty (no stent)	HTN/Obesity/ex-smoker	Outpatient follow up: angina-free
[24]	78 M	47	2 years	MI	Prednisolone	PCI : balloon angioplasty and thrombectomy	DM/HTN/Dyslipidemia	Two years later, total thrombotic occlusion of LCx
[25]	64 M	13	N/A	MI	Steroid	PCI : bare metal stent then thrombectomy for stent thrombosis	HTN/Obesity/ex-smoker	Sixth day of hospitalization he developed stent thrombosis due to premature stop of ASA
[26]	64F	236	15 years	MI	Romiblostim	PCI : stent then thombus aspiration. Eptifabitide and DAPT	Smoking / HTN	She developed partial stent thrombosis two days after discharge
[27]	81 F	34	1 year	MI	Non	PCI : stent in RCA + aspirin desensitization	Asthma / Arthritis / Aspirin allergy	Three months follow up showed stable PLT count and symptoms free
[28]	52 F	10 000	Long-standing	MI	Steroid / IVIG / rituximab	Optimized medical treatment, PCI not done	HTN/Seizure/mild CAD	Discharge
[29]	23 F	35	4 years	MI	Steroid	PCI with stent then DAPT	non	Discharged after five days without complications
[30]	63 M	50	19 years	MI	Prednisolone 5 mg	PCI with stent then DAPT (aspirin and ticlopidine)	HTN	Progressive narrow of RCA from 20% to 40%
[31]	67 M	<10	Chronic	ACS	Steroid, PLT transfusion, danazol, IVIG	IV nitrate, nicardipine then beta blocker, amlodipine and lisinopril (ASA not given)	CAD,PE,traumatic splenectomy, sarcoidosis,hypothyroidism	Discharged
[32]	36 F	32	8 years	Stroke	Steroid	Clopidogril 75 mg	non	Two months later she was diagnosed with another multiple left MCA ischemic stroke. One year later she was admitted again with left anterior cerebral artery
[33]	58 F	347	6 years	Stroke	Splenectomy 6 years ago, IVIG 5 days before the event	Aspirin and atorvastatin	HTN / DM / Dyslipidemia	Headache and vertigo recovered gradually without complications
[34]	31 F	normal	3 days	Stroke	PLT transfusion steroids	Antiplatelets therapy	None	Improved, discharged from the hospital three weeks after admission
[35]	33 M	84	New	Stroke	Non	Intraarterial thrombolysis followed by one day heparin	non (PFO on Echo but no DVT)	Twenty months later developed right basal ganglia and temporal lobe stroke
[36]	69 M	2	New	ACS	Prednisolone , IVIG	Not treated as he had only ECG changes without chest pain	HTN / DM	He suddenly collapsed and died of cardiogenic shock
[37]	55 M	42	Chronic	MI	Steroid IVIG responsive	IVIG steroids PCI	smoker HTN Dyslipidemia	Six years later had NSTEMI one day after receiving dexamethasone and rituximab for ITP relapse
[37]	61 M	322	10 years	MI	Steroid , splenectomy	PCI	smoker	Four years later patient was readmitted with a relapse of ITP
[38]	61 M	105	5 years	MI	Splenectomy, steroid , Eltrombopag	PCI with bare metal stent then DAPT	non	Discharged, no relapse for ITP during one year follow up
[39]	78 F	10	New	MI	IVIG	Nitrate to control angina, furosemide and digoxin	HTN, angina	Symptoms controlled by nitrate but he developed anteroseptal MI complicated by clinical signs of left ventricular failure
[40]	42 F	35	22 years	MI	Prednisolone, azathioprine. IVIG complicated with DVT	PCI with a stent for DAPT for 28 days, after the second STEMI she was shifted to Ticagrelor for 1 year.	prediabetes	On the night of discharge, she came back with in-stent thrombosis treated with thrombus aspiration then stent again
[41]	32 F	49	Chronic	Left vertebral a	Not mentioned	Not given anticoagulant or antiplatelet, only IVIG	not mentioned	Improved, three months later MRA complete resolution of cerebellar infarct
[42]	65 M	80	1 month	MI	Prednisolone	PCI with DES to proximal LAD	not mentioned	Discharged on DAPT with no symptoms or bleeding complications
[42]	67 M	12	10 years	MI	Splenectomy 10 years ago	CABG and discharged on clopidogril	not mentioned	Discharge with no symptoms or bleeding complications
[43]	49 F	54	20 years	MI	Splenectomy 7 years ago	ASA/Clopidogril, IV heparin, Tirofiban intracoronary then IV tirofiban infusion. PCI not done	not mentioned	Discharged
[44]	22 F	14.96	1.5 years	Stroke	Prednisolone, splenectomy with AV replacement (scheduled)	Aortic valve replacement, oxygen support, nitroglycerin infusion and morphine, intermittent BiPAP	bicuspid aortic valve pregnancy	Developed surgical site infection at the sternum then discharged
[45]	66 M	110	Recent	3VD	Prednisolone, IVIG	Staged PCI (RCA and LCX initially, 1 month later LM and LAD steted)	HTN / Hypercholesterolemia / Smoking	Discharge
[46]	53 M	50	New	MI	N/A	PCI DAP then steroidds IVIG romiplastin	DM dyslipidemia	20 days after his discharge, readmitted to the Hematology clinic due to lung infection and died from sepsis
[47]	60 F	26	6 years	MI	Steroid	PCI with thrombectomy with bare metal stent, DAPT for 1 month then ASA	DM/HTN/Dyslipidemia/family Hx. of CAD	After four months, the patient was completely asymptomatic
[48]	36 M	6	3 years	Stroke	Chinese patent drug	Eradication therapy for H. pylori	Non	Ddischarged home
[49]	48 M	41	New	MI	Newly diagnosed	PCI, LAD stent, DAPT for 1 year then ASA lifelong	HTN/DM/Hypothyroidism	Remain asymptomatic, treadmill after 18 months was negative for ischemia
[50]	44M	46	Chronic	MI	In remission not on treatment	Aspirin clopidogrel tPA PCI	Smoking	Discharged after five days of hospitalization
[51]	69 F	320	4 years	Skin	Eltrombopag 37.5 mg/day	Warfarin and clopidogril	DM / HTN	Ischemic toes salvaged by conservative therapy including hyperbaric oxygen therapy
[52]	61 F	68	Chronic	MI	N/A	PLT and packed RBC transfusion CABG	N/A	A stress test six months postoperatively showed no evidence of ischemia
[53]	37 M	8	New	MI	N/A	Splenectomy then CABG	N/A	Discharged, he was free from angina pectoris and the tendency to bleed
[54]	69 M	63	New	MI	N/A	Steroid then IVIG	Not mentioned	N/A
[55]	64 M	18	3 years	3VD	Not on treatment	Steroid then IVIG, CABG	Not mentioned	Elective splenectomy eight months after his cardiac surgery, continues to do well.
[56]	72 M	19.3	5 years	3VD	Steroid	Steroid, increased IVIG PLT transfusion	Smoker COPD hypercholestremic leg claudication DM	Six months after the surgery patient was alive and well
[57]	59 M	88	1 year	Unstable angina	Prednisolone	Steroid, IVIG, fresh whole blood, PLT transfusion during the CABG	Inferior mI	Discharged
[58]	60 F	42	6 years	MI	Managed expectantly	PCI	UC	Discharge on the fifth postoperative day
[59]	49 M	41	2 years	MI	Steroid	IV unfractionated heparin, clopidogrel, aspirin. (PTCA) and abciximab	IHD	No further chest pain, transferred back to his referring hospital
[60]	61 F	4	20 years	MI	Prednisolone	PCI	No PMH	Follow up CAG showed 95% focal in-stent restenosis in the BMSs site
[61]	62 F	3	N/A	Unstable angina	N/A	PTCI	HTN	N/A
[62]	54 F	7	4 months	Stroke DVT	Splenectomy, steroid	Platelet transfusion, enoxaparin fot DVT	Non	On day 6 developed thrombosis of the left mid-distal superficial femoral and popliteal veins."
[63]	63 M	2	New	Stroke	N/A	IVIG steroids	HTN	He was released
[64]	72 F	22	1 year	Mural thrombus (thoracic, abdominal aorta, and the common iliac)	Eltrombopag	Steroids IVIG rituximab eltrombopag	DM HTN dyslipidemia	Neurological disturbances remained, transferred to a rehabilitation hospital

**Table 2:** Characteristics of patients with ITP during the venous thrombotic events. LMWH, low molecular weight heparin; AF, atrial fibrillation; HTN, hypertension; DVT, deep vein thrombosis; DM, diabetes mellitus; ICU, intensive care unit; IVIG, intravenous immune globulin; PE, pulmonary embolism; ITP, immune thrombocytopenic purpura; PMH, past medical history; SSS, superior sagittal sinus; CVT, cerebral venous thrombosis

Reference	Age gender	PLT count at diagnosis of thrombosis x109/L	Duration of ITP	Site of thrombosis	Treatment before the event	Intervention for thrombois	PMH	Outcome
[65]	18 M	33	New diagnosis	SSS thrombosis	Non	Mannitol / Levetiracetam (no anticoagulation for low PLT)	Non	Discharged , became independent in most of the daily activity
[66]	48 F	187	3 years	Renal vein thrombosis, PE	Steroid, Danzol, Eltrombopag	Rivaroxaban thrombolysis	Non	Discharged ,with resolution of thrombus and embolus
[67]	19 F	Not mentioned	2 years	Portal vein thrombosis, mesnteric vein thrombosis	Not mentioned but finally splenectomy, she developed thrombosis in D21post OP	anticoagulation with warfarin for 1 year	Not mentioned	After three procedures she was left with 45 proximal and 10 cm of distal small bowel
[68]	38 F	950	Chronic (not mentioned duration)	Portal vein thrombosis, bowel infarction	Steroid and IVIG, splenectomy 9 days before the event	Started on ASA then on the 4th day started on warfarin	Non	6 months later doppler revealed revcanalization so anticoagulation stopped
[69]	83 M	29-37	1.5	DVT in the right thigh	Tranexamic acid for hematoma, no mention of which treatment was given for ITP	Urokinase infusion for 8 days	Non (only trauma-induced hematoma 1.5 years ago)	Thrombosis dissolved, no recurrence of DVT
[70]	56F	14	2 months	DVT (left femoral) and PE	Steroid for 2 months then IVIG 3 days before the event	LMWH then warfarin	Non	Complete resolution of PE on CT after 3 weeks, complete resolution of LL edema
[71]	55 F	124	20 years	Cerebral venous sinus thrombosis	Steroids, IVIG, Romiblostim, eltrombopag prednisolone	Heparin then warfarin	Not mentioned	Resolving of symptoms and PLT 78 000
[72]	39 F	32	Chronic (not mentioned duration)	Cerebral venous sinus thrombosis	Eltrombopag (not compliant)	Heparin infusion then warfarin	DM	After 2 weeks of treatment, developed a new right anterior frontal small hemorrhagic infarction
[73]	54 M	33	Newly diagnosed	Bilateral DVT with PE	Non	Heparin then dabigatran 150 BID	Non	1 week later he developed PE, he was shifted to warfarin but he developed hepatotoxicity, and switched to Rivaroxaban
[74]	40 F	20	8 years of low PLT but not labeled as ITP	Right brachial and axillary vein, and distal brachial, radial and ulnar arteries	Non	Above elbow amputation or right upper limb then oral warfarin overlap with enoxaparin	2 episodes of LL DVT	1 month later presented with a right basal ganglia infarct
[75]	44 F	160	1 year	Right jugular vein, right subclavian vein sigmoid and transverse cerebral sinus	Steroids , prezolon, IVIG and eltrombopag, romiplostim	N/A	Hypothyroidism smoking	Complicated by epidural hematomas and died in ICU
[76]	26 F	311	4 years	Intracardiac thrombus and thrombus in pulmonary artery	Steroid (resistance) then splenectomy 1.5 years ago. IVIG, Eltrombopag, danazol, vincristine	Warfarin	Non	N/A
[77]	26 M	65	One month	CVT, SSS, and right transverse	Prednisolone IVIG	Warfarin dexamethasone	N/A	Discharged after this admission
[78]	77 M	80	Chronic (not mentioned duration)	Thrombus of left atrial appendage occluder	Splenectomy long time ago, Eltrombopag 1 month before the event	Refer for surgical left atrial appendage closure and AV replacement with a bioprosthesis	AF, DM, HTN, moderate to severe aortic stenosis	N/A

Among patients with ITP with known duration (n=47), the mean duration of ITP to thrombotic events was 7.58 years, with the most prolonged duration being 30 years. The mean time for the development of venous thrombosis was 4.9 years, while for arterial thrombosis was 8.12 years. The mean PLT count during the time of thrombosis was 90.2 x 109/L in 75 patients, the mean PLT for patients with venous thrombosis was 156.7 x 109/L, while those with arterial thrombosis were 77.1 x 109/L. The lowest PLT associated with thrombosis was 1 x 109/L, and the highest PLT was 658 x 109/L. However, most patients (n=66) had thrombosis with a PLT below 100 x 109/L, and 50 patients had PLT below 50 x 109/L. Seven events happened while being treated with IVIG, five were arterial events, and two were venous thrombotic events.

Regarding comorbidities, 32 patients had no significant past medical history (PMH) or were not reported. The most commonly reported medical condition was hypertension in 23 patients, followed by diabetes mellitus in 16 patients and prediabetes in one patient, and dyslipidemia in eight patients. Cardiac valve defects in four patients and asthma in one patient, smoking in nine patients, and coronary artery disease in seven patients. Among all patients, mortality was reported in five patients from complications related to thrombosis or infarction. Some 12 patients had developed another complication after admission; another thrombotic event or infarction. Among them, seven had cardiac thrombotic events, including ischemia and stent thrombosis, three cerebrovascular events, and one had venous thromboses.

Discussion

Thrombosis is a process characterized by complex pathophysiology. Generally, thrombosis occurs when one or more of Virchow’s triads are present; blood stasis, endothelial injury, or hypercoagulable states. In arterial thrombosis, the main culprit is endothelial injury, while in venous thrombosis, the etiology can be explained by the stasis of blood in veins and procoagulant states are the underlying factors favoring thrombosis. ITP commonly presents with bleeding and paradoxically sometimes it presents with thrombosis [3]. To understand the link between the two paradoxical processes, the existing data and evidence suggest an increased incidence of thromboembolism in patients with ITP [79]. However, the role of patient characteristics including age, gender, duration of ITP, and treatments used and the hematological parameters at the time of the event, was not mentioned before in previous systematic reviews [80] and other studies included patients based on diagnosis from the code without elaborating the inclusion and exclusion criteria [81].

Although there is a slightly higher incidence of ITP in young females [82]. Our review found out that among the patients with ITP, both genders are prone to develop paradoxical thromboembolic complications with slightly higher numbers in males. But considering that coronary artery disease is more common in males, it is expected to have males being more affected by cardiac events [83] including patients with ITP (Table 3). This supports that gender has no significant role in the pathogenesis of thrombosis.

**Table 3:** Gender distribution for ITP patients with thrombosis or infarction. ITP, immune thrombocytopenic purpura

	Male	Female
Arterial thrombosis	34	29
Venous thrombosis	5	9

It is clear that in ITP, a low PLT is not protective against thrombosis and infarction. In this review, the data show that thrombosis can occur in all stages of ITP, including patients with a new diagnosis, persistent, and chronic ITP (with the persistent stage being the least risky phase); this includes both patients on treatment and patients who were managed expectantly. This suggests that the ITP itself has the potential for being a prothrombotic state. Additionally, among ITP patients who developed thrombosis, a large percentage of them had developed a second thrombotic event after admission or discharge. This suggests that ITP is a disease that carries not only a prothrombotic state but with a significant risk of recurrence or complications as 13/78 patients develop a second event. Additionally, many patients had both arterial and venous thrombotic events making ITP a rare cause of arterial and venous thrombosis as in patients number [63, 76]. Although most patients who had events had PLT above 100, there is no clear-cut number of platelets that are safe to prevent thrombosis in patients with ITP; as thrombosis was observed in patients with low PLT as well low as 1 x 109/L. However, the presence of other factors predisposing to infarction and thrombosis, including age-advanced atherosclerosis, uncontrolled blood pressure, and diabetes, could have marked effects [84].

Patients with ITP have low platelets and most guidelines recommend avoiding the use of antiplatelet agents or anticoagulation when PLT is less than 50,000 x 109/L. The major difficulty is that there is no anticoagulant that can treat thrombosis without also increasing the chance of bleeding. Therefore, patients with ITP with thrombosis are difficult to manage and there is no unified treatment plan in such a situation; although, many experts recommend platelet transfusion to increase PLT to a safe level and then to give anticoagulation or antiplatelet.

Limitations

This review focuses on studying the impact of patient characteristics, disease course, and treatment modalities on the incidence of thromboembolism in patients with ITP. As such events are rare; mainly case reports have been studied with no control population and a very small sample size of 73 articles.

Theory

There are many theories that can explain unexpected thrombosis and infarction in patients with ITP. First, persistent activation of the immune system leads to accelerated atherosclerosis as in other autoimmune conditions, predisposing the patient to arterial thrombotic events [85]. Interestingly, platelet microparticles (PMPs) have been found to play a significant role in thrombus development in ITP. Platelet microparticles are minute vesicles formed by platelet membranes that are undetectable during routine platelet counting and are usually produced in association with platelet activation [86]. PMP levels were found to be higher in ITP patients compared to a control population without ITP in many studies, and they were also proven to be protective against hemorrhage. PMPs, in excess, can increase the synthesis of thrombin. They are hypothesized to play a function in clot formation as a result [87]. At the moment there is no specific treatment that can target the platelet microparticles. The treatment used is another probable cause for thrombus development in ITP. First, IVIGs can cause thrombosis by raising blood viscosity [88] and thrombin generation, as well as by directly influencing the vascular endothelium, which results in higher amounts of von Willebrand factor (vWF) antigen. Some studies showed elevated levels of vWF in ITP patients, particularly patients with long-standing diseases. Additionally, thromboelastography showed a relatively higher thrombotic tendency correlating to elevated levels of the vWF antigen levels [89]. Thrombopoietin receptor agonists are newer agents added to ITP treatment almost in the last decade. These are platelet growth factors that act on megakaryocytes and megakaryocyte precursors in the same way that endogenous thyroid peroxidase (TPO) does, boosting their growth, differentiation, and enhancing platelet production [90]. Elevated PLT beyond the target level is an expected side effect that probably plays a pivotal role in raising the risk of thrombotic events in patients treated with thrombopoietin receptor agonists. Despite that, thrombotic events have been reported with PLT that is lower than normal in patients treated with TPO-Ras, favoring the fact that megakaryocyte activation itself leads to an increased risk of thrombosis prior to the rise in PLT [91]. Additionally, manufacturers recommend using the lowest minimal dose to keep PLT above 50 x 109/L and not to aim for normal PLT. This supports that platelets in ITP are active, and patients rarely report bleeding compared to patients with the same count in other diseases [92]; this may be related to younger platelets with more hemostatic effect. Observational studies of ITP patients treated with thrombopoietin receptor agonists have revealed a modestly higher rate of thrombosis [90, 93]. The data showed that nine patients had treatment with eltrombopag; all of them had PLT above 100 x 109/L at the time of the thrombosis except one patient who had a PLT of 22 x 109/L. This emphasizes the need for frequent monitoring of PLT in patients on TPO-Ras to avoid the rise of PLT above the target and subsequent development of thrombosis. However, it is important to note that three of the nine patients who were on TPO-Ras had a splenectomy because splenectomy can result in an increase in the number of active circulating platelets and prolong their lifespan which can contribute to thrombosis.

## Conclusions

Although patients with ITP are prone to life-threatening bleeding, it is crucial to know that ITP patients are susceptible to thromboembolic phenomenon. These events can occur at any stage of the disease in both patients on active treatment and those not on medications and with various PLT. All patients with chronic active ITP treated with IVIG or TPO-RA should be observed closely for any thromboembolic events. The question of thromboprophylaxis use despite low PLT, especially if no active bleeding, is yet to be answered and needs further studies and trials. We recommend, ITP patients to be evaluated for the risk of thrombosis and atherosclerotic disease to avoid difficult situation where patient has low PLT and he or she requires anticoagulation.
